# *tmem-258* is dispensable for both nuclear anchorage and migration in *C. elegans*

**DOI:** 10.17912/micropub.biology.000208

**Published:** 2020-01-02

**Authors:** Ellen Faith Gregory, Daniel Aaron Starr

**Affiliations:** 1 University of California, Davis

**Figure 1:**
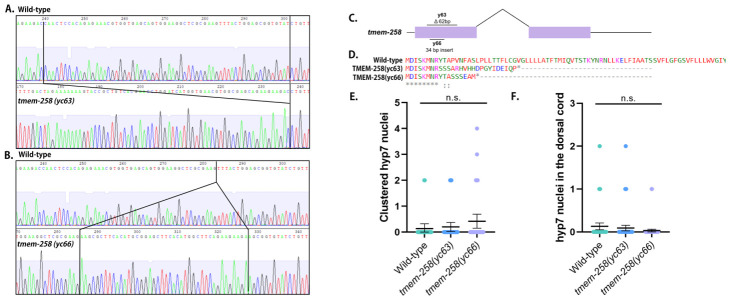
Trace files comparing wild type to **A.** *tmem-258(yc63)* and **B.** *tmem-258(yc66)* mutations. **C.** *tmem-258(yc63)* is a 62bp deletion, and *tmem-258(yc66)* is a 34bp insertion. **D.** Both mutations are predicted to create early stops in TMEM-258. **E.** The number of syncytial hyp7 nuclei clustering together (n=50). **F.** The number of mislocalized hyp7 nuclei in the dorsal cord (n=100). Error bars are 95% confidence interval.

## Description

Human TMEM258 is involved in stress pathways and has been implicated in inflammatory intestinal disorders (Graham et al., 2016). The *Drosophila* ortholog Kuduk consists of a transmembrane and an intramembrane domain and localizes to the outer nuclear membrane, where it regulates myonuclear positioning and morphology (Ding et al., 2017). Kuduk is the first characterized cytoplasmic regulator of the LINC (linker of nucleoskeleton and cytoskeleton) complex. LINC complexes are conserved throughout eukaryotes and consist of SUN (Sad-1 and UNC-84) proteins, which localize to the inner nuclear membrane and bind to lamin, and KASH proteins (Klarsicht, ANC-1, Syne homology) that span the outer nuclear membrane and interact with cytoskeletal elements. KASH and SUN bind in the perinuclear space to bridge the nucleus and cytoskeleton and transfer force across the nuclear envelope (Starr, 2009). *Drosophila* Kuduk has been shown to help anchor the nucleus in place via the LINC complex, but the molecular mechanisms it uses to carry out nuclear migration remain unknown.

We hypothesized that *tmem-258* (formally Y57E12AM.1) is required for nuclear positioning and migration in *C. elegans*. We used CRISPR-Cas9 to introduce non-specific mutations in *tmem-258* and generated two frameshift mutants, *yc63* and *yc66,* which created early stop codons (Fig. 1A-D). We then assayed nuclear migration in two different tissues. First, nuclei migrate across the dorsal midline in hypodermal (hyp7) precursors during normal *C. elegans* embryogenesis (Fridolfsson et al., 2018). If hyp7 nuclear migration fails, ectopic nuclei can be seen in the dorsal cord. For example, in an *unc-84(n369)* null line, an average of 15.0 nuclei were abnormally in the dorsal cord (Malone et al 1999). Neither *tmem-258(yc63)* nor *tmem-258(yc66)* animals exhibited mislocalized nuclei, suggesting hyp7 nuclear migration was successful (Fig. 1F). Second, we quantified P-cell nuclear migration in L1 larvae. P-cell nuclei migrate from the lateral to the ventral side of the worm where they divide and differentiate to form the vulva and GABA neurons. At 25˚C, *unc-84(n369)* mutants typically are missing 6.5±0.60 GABA neurons (Bone et al., 2016). To determine whether *tmem-*258 mutants enhance the P-cell nuclear migration defect of *unc-84*, we crossed *tmem-258(yc63)* worms to *unc-84(n369)* and quantified P-cell nuclear migration failure at 15°C by counting GABA neurons with UNC-47::GFP. The *tmem-258(yc63); unc-84(n369)* double mutant were missing an average of 0.70±0.3 (mean ± 95% CI, n=25) GABA neurons, equal to the 0.60±0.4 GABA neurons of the *unc-84(n369)* single mutant at 15˚C. Finally, we tested whether *tmem-258* is required for nuclear anchorage in *C. elegans* by counting the number of syncytial hyp7 nuclei clustering together in adults. The *tmem-258* mutants had no significant nuclear anchorage defects (Fig. 1E). We therefore conclude that *tmem-258* is not required for nuclear positioning in *C. elegans* hyp7 cells.

## Reagents

The *tmem-258* mutations were generated using the guide sequence 5’-TCGCGAAGTTTACTGGAG-3’ injected into UD398: *him-8 (e1489) ycIs10 [pBS sk+; pSL589]* animals containing a hyp7 GFP nuclear marker. Injections were done with *dpy-10* co-CRISPR according to previous methods (Paix et al., 2015). *tmem-258(yc63)* corresponds to UD600 and *tmem-258(yc66)* is UD609. *tmem-258(yc63)* was crossed to UD87: *unc-84(n369); oxIs12[unc-47::gfp, dpy-20(+)]; ycEx60[odr-1::rfp]* to create UD623: *tmem-258(yc63), him-8(e1489) ycIs10; unc-84 (n369) oxIs12[unc-47::gfp] ycEx60[odr-1::rfp unc-84(+)]* which was used to assay P-cell nuclear migration.
